# Progress in mechanism-based diagnosis and treatment of tuberculosis comorbid with tumor

**DOI:** 10.3389/fimmu.2024.1344821

**Published:** 2024-01-17

**Authors:** Chuan Wang, Rong-Qi Zou, Guo-Zhong He

**Affiliations:** ^1^ School of Public Health, Kunming Medical University, Kunming, China; ^2^ Vice Director of Center of Sports Injury Prevention, Treatment and Rehabilitation China National Institute of Sports Medicine A2 Pangmen, Beijing, China

**Keywords:** tuberculosis (TB), tumor, cancer, tuberculosis comorbid with tumor (TCWT), pathogenesis, immunotherapy

## Abstract

Tuberculosis (TB) and tumor, with similarities in immune response and pathogenesis, are diseases that are prone to produce autoimmune stress response to the host immune system. With a symbiotic relationship between the two, TB can facilitate the occurrence and development of tumors, while tumor causes TB reactivation. In this review, we systematically sorted out the incidence trends and influencing factors of TB and tumor, focusing on the potential pathogenesis of TB and tumor, to provide a pathway for the co-pathogenesis of TB comorbid with tumor (TCWT). Based on this, we summarized the latest progress in the diagnosis and treatment of TCWT, and provided ideas for further exploration of clinical trials and new drug development of TCWT.

## 1 Background

In recent years, the stubbornly high global incidence of TB and tumor renders the two as public health problems that threaten human health and as major factors in the global disease burden (1–3). In the past two decades, the number of new TB patients stays basically high. It is estimated that the year 2022 saw 10.6 million new TB patients in the world, with an incidence rate of 133/100,000 (3). TB is also the world’s leading cause of death from a single infectious source, with 1.3 million deaths globally in 2022 (3). There are many epidemiological evidences showing that TB coexists with tumors, where TB is a susceptible factor for tumor, and tumors can augment the incidence risk of TB (4–6). TB is attributed to an increased risk of cancer mortality, and TB patients have higher comorbidity and mortality with tumor (7, 8). Many epidemiological evidences show that TB is closely related to tumors, and the risk of TB patients comorbid with lung cancer, pleural mesothelioma, Hodgkin’s lymphoma and other cancers is higher than that of the normal population (5, 9). Studies (10) have shown that cancer itself is an independent risk factor for developing active mycobacterium TB infection, however, this risk varies greatly among different cancer types, with lung cancer and hematologic malignancies having a higher risk (11, 12).

TB and tumor are bidirectional related diseases, and there is a causal relationship between them. TB facilitates the occurrence and development of tumors, and tumor causes TB reactivation, and there is a symbiotic relationship between the two. Studies have shown that both tumor patients and TB patients are prone to generate autoimmune stress reactions to the host immune system. With similarities in immune response and pathogenesis, TB and tumor can facilitate disease progression and anti-tumor or elimination of mycobacteria through immune cell reactions such as T cells (13, 14). Immunotherapy targeting T cells has also been applied to the treatment of TCWT (15). In this paper, a systematic study was conducted on the disease risk, mechanism, diagnosis and treatment of patients with TCWT, and the relationship between the two disease mechanisms was clarified to provide clinical decision-making basis for the treatment of TCWT.

## 2 Prevalence trend of TCWT

**Figure 1:**
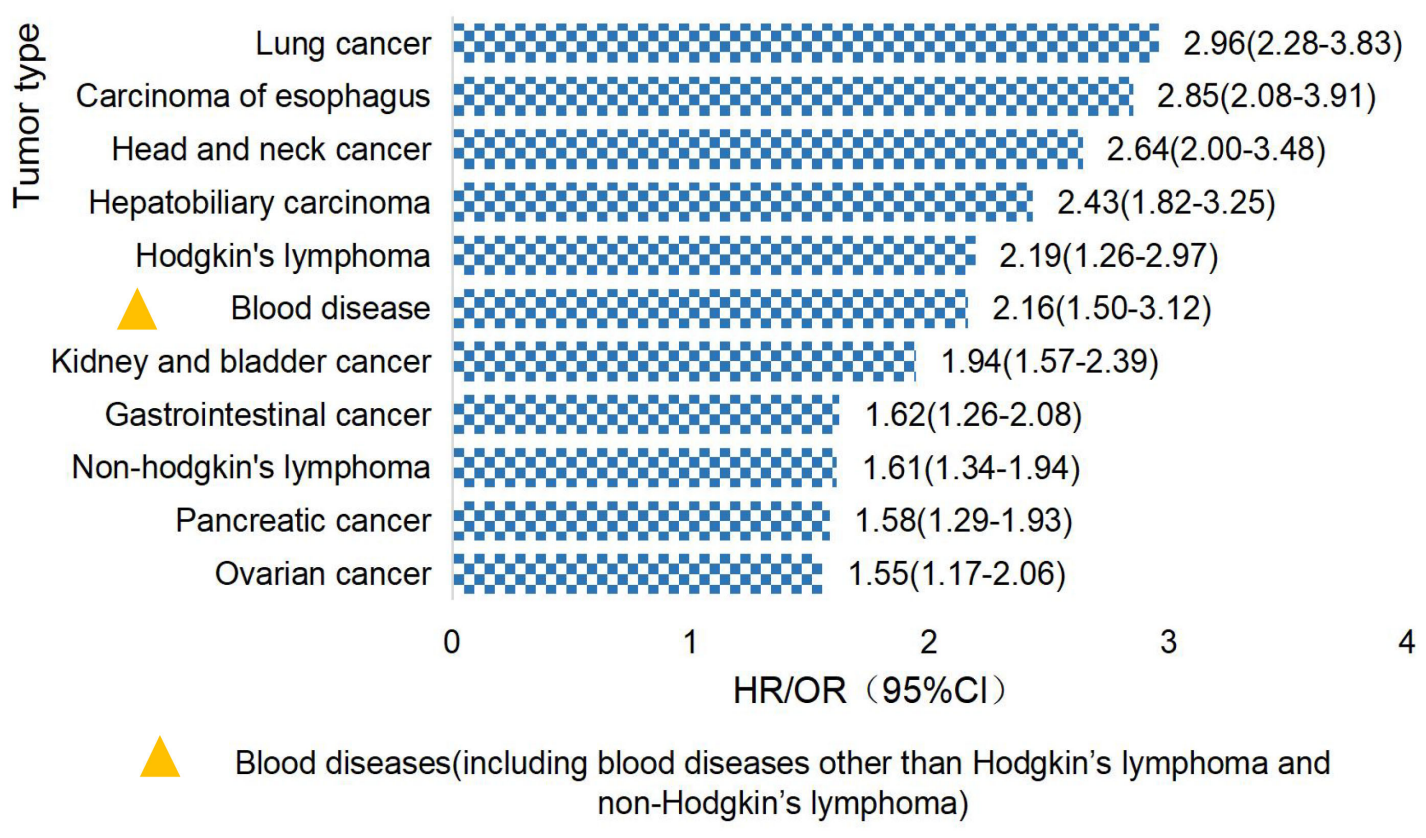
Top 10 TB-attributed cancer risks in European and American countries.

**Figure 2:**
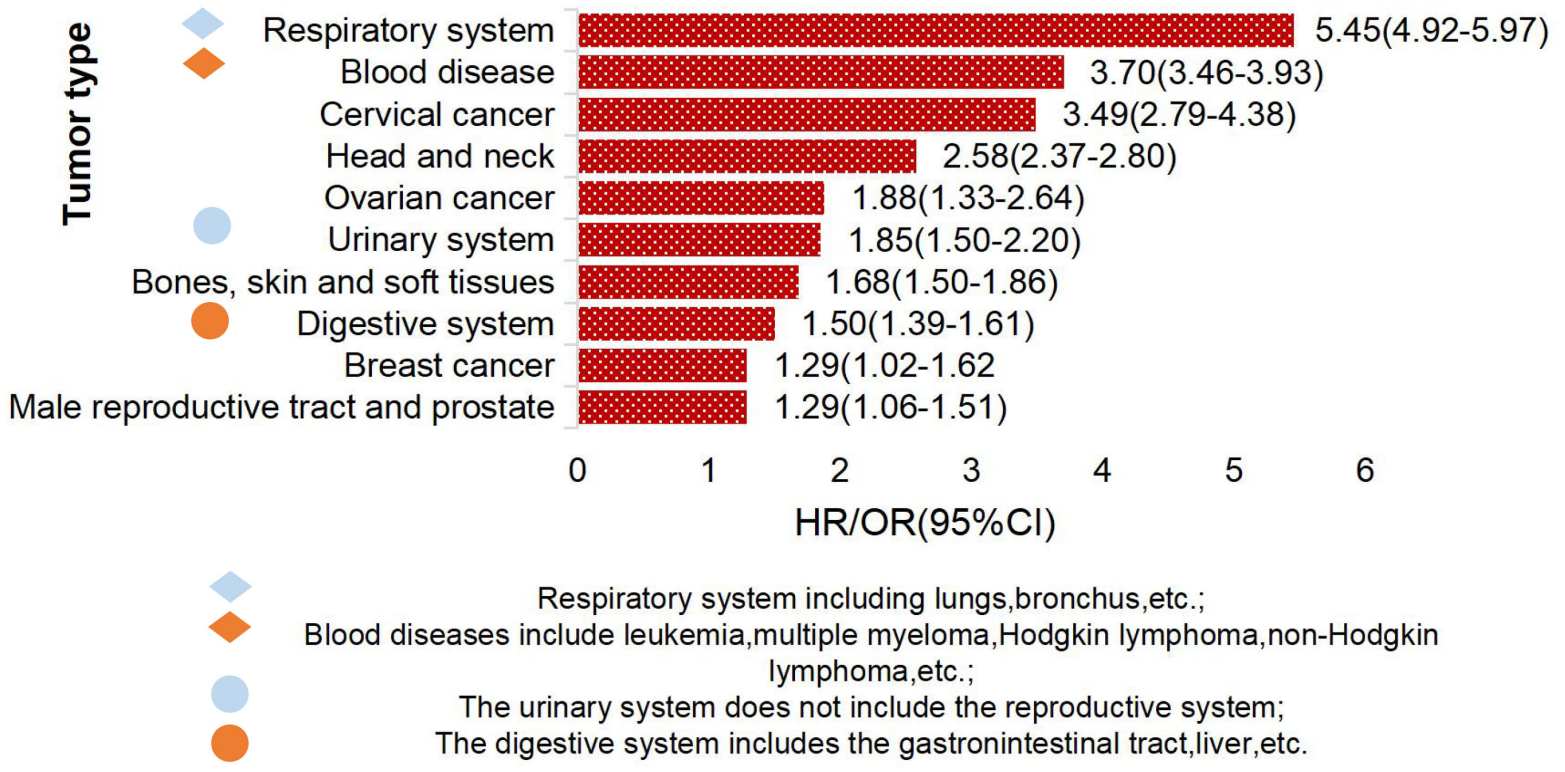
Top 10 TB-attributed cancer risks in China.

Studies have found that TCWT is on the rise, with 2.33% of global cancer (tumor) incidence attributed to TB (6, 16, 17). Studies have shown that TB patients have a high risk of developing cancer (18). The overall incidence of cancer induced by TB was 1.60(95%CI, 1.28-2.01) (19), and the incidence of lung cancer was higher in TB patients, which was about 3.0 (95%CI,2.35-3.32) (20). TCWT boosted mortality risk, with standardized mortality rates of respiratory cancer, blood cancer and head and neck cancer being 5.45, 3.70 and 2.58, respectively (21). The risk of TCWT varies in different regions. In European and American countries, the risk probability of TCWT mainly includes lung cancer, esophageal cancer, head and neck cancer, hepatobiliary cancer, Hodgkin’s lymphoma, etc. (17, 22–24), as shown in **Figure 1**. In Asian countries represented by China, high-risk tumors of TB patients are mostly manifested in diseases of respiratory system, blood system, cervical cancer, head and neck tumors and others (18, 25, 26), as shown in **Figure 2**.

TB is associated with the pathogenesis of tumors, and can lead to tumor occurrence, showing a positive correlation (23). Meta-analysis has shown that TB is an independent risk factor for cancer, and the risk of cancer in TB patients is significantly increased compared with the normal population (27). A retrospective study showed that of 3776 TB patients followed up, 86 (2.3% of the population) developed lung cancer (28). A Danish national cohort study (29) showed that TB patients had an increased long-term risk of developing cancer compared to the general population. There is a 50% increased risk of lung cancer two years after TB (9). A long-term follow-up study in Taiwan suggested that the proportion of malignant tumors in newly diagnosed TB patients increased year by year, from 9% in 2005 to 13% in 2015 (18).

Tumor has an activation effect on TB, and immunotherapy in tumor patients will cause the latent mycobacterium TB to reactivate, promote the continuous proliferation of mycobacterium TB in the body, and secrete immunosuppressive factors to inhibit the immune function of the body (30, 31). It causes a significant increase in the risk of active TB in tumor patients, with an incidence rate that is 9-13 times that in tumor free patients (11, 32, 33). A clinical study (34) showed that the median incidence of latent TB infection in patients treated with infliximab after antitumor therapy was 12.1 times higher than in patients treated with etanerceib. Multiple studies (35–37) have shown that cancer patients are at twice the risk of developing TB compared with the general population. Patients with lung cancer are 6 times more likely to develop TB than those without lung cancer (31). The risk of TB in gastric cancer patients was 2.63 times that of the general population (IRR 2.63, 95% CI 1.96-3.52) (38), and the risk of TB in adult patients with blood tumors was 3.3 times that of the general population (IRR 3.53; 95% CI 1.63-7.64) (12).

In summary, an array of epidemiological investigations have shown that TB and tumor have mutually promoting effects, and more and more epidemiological studies have proved the comorbid relationship between the two, suggesting that studies on the pathogenesis and molecular mechanism of the two should be reinforced to provide support for public health security and alleviate the global disease burden.

## 3 The mechanism of TB and tumors

### 3.1 Possible mechanisms of TB- induced tumorigenesis

#### 3.1.1 T cells

**Figure 3:**
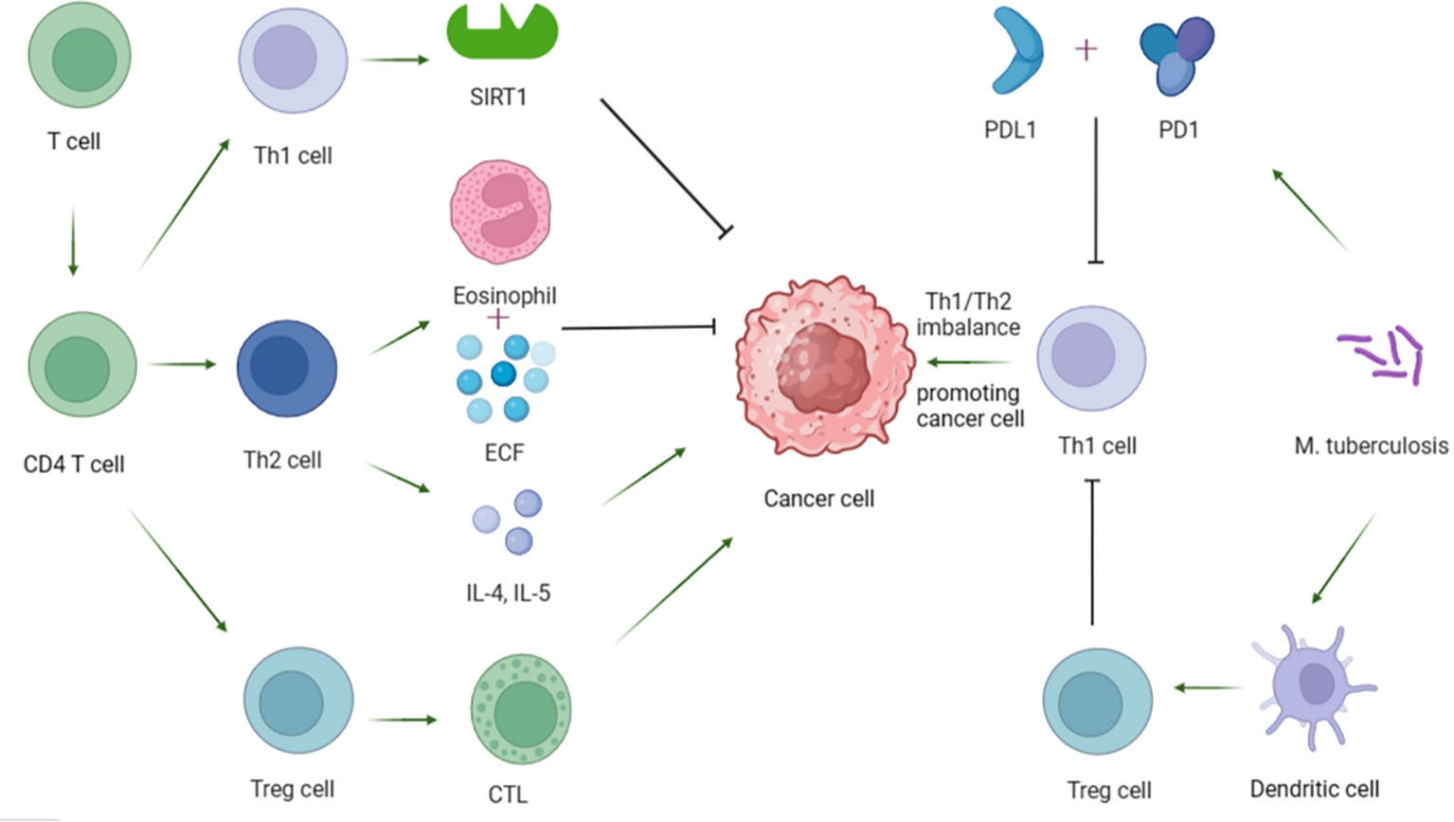
Mechanism of T cell influence on tumor cells and possible mechanism of MTB causing tumor.

T cells play a key role in regulating tumor growth and metastasis. Among them, CD4+ T cells can be differentiated into Th1, Th2 and regulatory T cells (Tregs) according to different functions and markers. Th1 regulates immune cell function by increasing the activity of silence-message regulatory factor-associated enzyme 1 (SIRT1) dependent on nicotinamide adenine dinucleotide (NAD), thereby exerting anti-tumor effects (39). Th2 is generally believed to play an anti-tumor role through the expression of eosinophilic and eosinophilic chemotactic factor (ECF), and some scholars have also proved that TH2 can play a pro-tumor role through the secretion of cytokines IL-4 and IL-5 (40). More researchers profess the importance of Th1/Th2 imbalance leading to impaired immune function and augmented escape of tumor cells, thus promoting tumor formation (41). At the same time, studies have shown that Th1/Th2 imbalance in peripheral blood is closely related to TB nosogenesis (42). TB patients show reduced Th1 response and/or enhanced Th2 response (43), and the severity of the disease is closely related to Th1 response, the lower the Th1 response, the more severe the disease (44). To delve into the potential mechanism, Cao et al. compared the immune response of T cells in patients with active pulmonary TB and mice treated with MTB and lung cancer cells, and proved that MTB may inhibit Th1 immune response through the programmed cell death protein 1 (PD-1)/programmed death ligand 1 (PD-L1) signaling pathway. This may lead to Th1/Th2 imbalance and further promote lung cancer metastasis (45). In recent years, studies on the pathogenesis of Tregs in cancer have also attracted the attention of many researchers (41). Tregs can protect tumor cells by inhibiting cytotoxic T lymphocytes (CTL) mediated apoptosis (39, 41, 46). Furthermore, Jie et al. found that Tregs was closely related to the occurrence of TB, and the results showed that the percentage of Tregs in peripheral blood of patients with active TB was significantly higher than that in the latent TB group or control group (Tregs: 11.44 +/- 2.69% vs. 7.54 +/- 1.56% vs. 4.10 +/- 0.99%, p < 0.05) (47). The possible mechanism is that MTB can enhance the polarization ability of Tregs by driving the high expression of BTLA in dendritic cells (48). The increase of Tregs activity can lead to immunosuppression and a decrease in the number of Th1 cells, which are conducive to the occurrence and progression of skin cancer (49). In addition, it has been proved that the production of Tregs and its inhibitory properties can also be regulated through the expression of PD-1 and the binding of PD-1 to PD-L1 (46). For this reason, the treatment of immune checkpoint inhibitors (ICIs) combined with PD-1 or PD-L1, especially for cancers such as non-small cell lung cancer, has shown remarkable efficacy, and may also be of great significance for the treatment of lung cancer caused by TB (46) (**Figure 3**).

#### 3.1.2 Myeloid derived suppressor cells

MDSC is a group of heterogeneous cells from bone marrow, which can inhibit immune cell response through reactive oxygen species (ROS) molecules and other pathways, and it has been proved by abundant evidences that it plays an important role in promoting tumor malignant growth and metastasis in tumor immunity (50–53). The reason: On the one hand, in the tumor microenvironment, MDSC exerts an inhibitory effect to produce excess ROS, while MDSC up-regulates the production of ROS in the high-concentration ROS environment. ROS can produce highly destructive hydroxyl radicals, resulting in the damage of DNA, proteins and lipids in the body. At the same time, oncogenes jun and fos are also activated, which eventually lead to the occurrence of tumors under the action of various factors (5, 54–56). On the other hand, in order to maintain an immunosuppressor environment, MDSC secretes a series of chemokines, thereby playing a role in the recruitment of Tregs and thus promoting the development of tumors (41, 46, 57). In addition to its role in tumors, MDSC has gradually been shown to play an important role in many chronic infectious diseases, especially in TB (58). Studies have shown (59) that under abnormal conditions of chronic infections such as TB, excessive production of MDSC will be caused to inhibit host protective T lymphocyte response. The possible mechanisms are as follows: First, MDSC changes the polarization direction of monocytes and macrophages by producing ROS, thereby inhibiting the anti-inflammatory response; On the other hand, studies have shown that MDSC inhibits the immune response of T cells and effector B cells by inducing cells such as Tregs (58). Therefore, it is very possible that MDSC is produced after infection with Mycobacterium TB, and MDSC ultimately promotes the occurrence and development of tumors through up-regulation of ROS and recruitment of Tregs.

#### 3.1.3 Macrophages

Tumor-associated macrophages (TAMs) are one of the major tumor-infiltrating immune cell types and are generally divided into one of two functional contrast subtypes, classically activated M1 macrophages and replacement-activated M2 macrophages. The former usually exerts anti-tumor functions, including directly mediated cytotoxicity and antibody-dependent cell-mediated cytotoxicity (ADCC) to kill tumor cells; the latter promotes the formation and metastasis of tumor cells by inhibiting the anti-tumor immune response mediated by T cells. When the body is exposed to MTB, macrophages play an important role as the main innate immune cells (60, 61). The relevant mechanism of action may be as follows: First, it has been found that IL-37 induces the polarization of macrophages towards M2-like phenotype during MTB infection (62, 63), which may further promote the occurrence and metastasis of tumor cells. Secondly, MTB toxic factor ESAT-6 can drive the polarization of macrophages towards pro-inflammatory M1 phenotype, and subsequently towards anti-inflammatory M2 phenotype, and can also drive the transformation of activated M1 phenotype into M2 phenotype (64), which may play a role in promoting tumor. In addition, in the process of inflammation, activated macrophages will gather at the site of MTB infection and produce a large amount of active nitrogen and ROS, etc. ROS promotes the occurrence and development of tumors by activating oncogenes jun and fos (54, 55). Some scholars used a mouse model infected with MTB to test the effect of macrophage depletion on the occurrence of lung cancer, and the results showed that the incidence of lung cancer in TB mice with macrophage depletion was significantly lower than that in control TB mice without macrophage intervention (65).

#### 3.1.4 Epidermal growth factor receptor signaling

**Figure 4:**
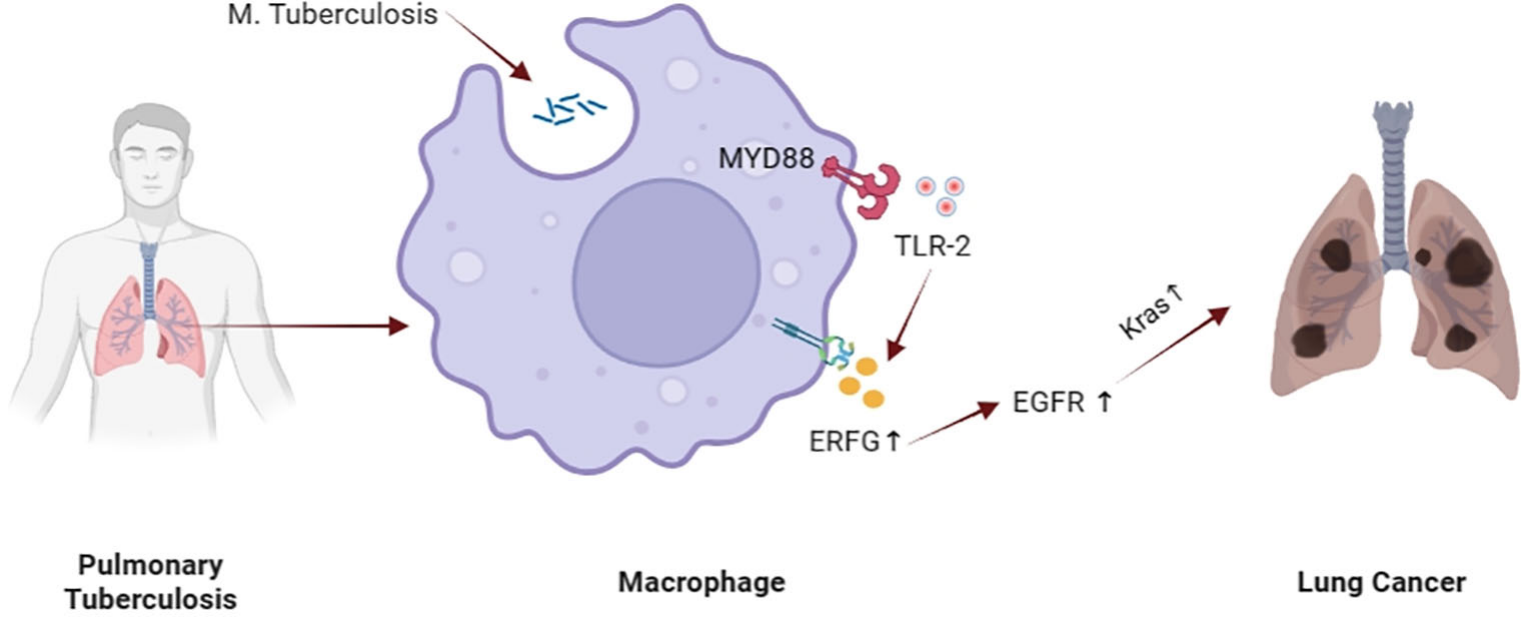
Possible mechanism of MTB causing tumor through EGFR pathway.

EGFR belongs to the ERBB family of tyrosine kinase receptors. The EGFR signaling cascade is a key regulator of cell proliferation, differentiation, division, survival, and cell proliferation, and has been shown to play an important role in regulating the proliferation, survival, and differentiation of tumor cells, and is highly expressed in more than 60% of Nonsmall Cell Lung Cancer (NSCLC) (66). In recent years, more and more researchers have provided evidence that MTB can cause tumors through the EGFR pathway: Nalbandian et al. found that MTB infected macrophages induce the production of highly efficient epidermal regulatory hormone (EREG), thus activating the EGFR signaling pathway through EREG and promoting cancer progression (5, 67). Meanwhile, studies have demonstrated in mouse models that MTB infection can stimulate the expression of EREG in a toll-like receptor 2 (TLR2) -dependent manner, and EREG binds to EGFR in membrane or soluble form to stimulate downstream signal transduction, thereby inducing activation and mutation of k-ras gene, leading to the occurrence of lung cancer (67, 68) (**Figure 4**).

In addition, EGFR mutations are often directly or indirectly associated with the pathogenesis of some cancers with high global morbidity and mortality, such as lung cancer, pancreatic cancer and head and neck cancer (69–73). Approximately 15% of Caucasian patients and 30%-40% of Asian patients with lung adenocarcinoma carry EGFR mutations (74). Among them, the two major classical mutations are exon 19 deletion and exon 21 L858R mutation, with the incidence of 44% and 41%, respectively (75). EGFR mutations are highly prevalent in tumors, and TB can cause high EGFR mutations. Luo et al. (70) found that patients with lung scar cancer or old TB had a higher incidence of EGFR mutation (p =0.018), especially exon 19 deletion (p<0.001), in patients with lung adenocarcinoma than those without TB focal points. This was also fully demonstrated in a retrospective study of nearly 500 patients with lung adenocarcinoma (76), with a higher frequency of EGFR mutations in the TB group than in the non-TB group (56% vs 34%, p=0.038). A study in Taiwan evaluated the correlation of EGFR mutation outcomes in 275 TB patients (77), with 191 patients (69.5%) having a high EGFR mutation in their study. Although it is certain that TB can induce EGFR mutation and increase the risk of tumor development, the exact molecular mechanism remains to be further elucidated. TB not only affects the mutated status of EGFR, but also affects the treatment response of patients treated with EGFR tyrosine kinase inhibitors (TKIs). At present, EGFR-TKI targeted therapy has become the standard first-line treatment for patients with advanced EGFR-mutated NSCLC, which can significantly improve their prognosis (78). Simultaneous use of antituberculosis drugs and TKIs in a comorbidities patient for EGFR-mutated lung cancer patients with active TB has shown a safe and alternative treatment strategy (79).

### 3.2 Possible mechanisms of tumor-induced TB

#### 3.2.1 Tumor effect

Cancer is largely regarded as a disease caused by gene mutation and gene alteration, and the metabolic changes of tumor cells driven by oncogenes can limit the immune response of the body by affecting the tumor microenvironment (TME), thus causing serious adverse effects on the normal immune defense function of the body (80, 81). At present, the mechanism of TB induced by tumor itself is not completely clear. The possible mechanisms are as follows: First, there is a high level of adenosine triphosphate (ATP) in TME, and CD73 decomposes ATP through dephosphorylation, and finally produces adenosine (82). Adenosine has a significant inhibitory effect on immune response (83) and can also play a protective role in extracellular bacteria and fungi, including MTB, by mediating type 3 immune effects (84). In addition, studies on lung cancer patients and mouse models have proved that lung cancer can lead to the loss of microbial diversity, the reduction of the total amount of bacteria and the change of bacterial composition, which can lead to the imbalance of microbial flora, resulting in the reduction of the stability of the body’s immune homeostasis, and thus increase the host’s susceptibility to various pathogens (85, 86). Studies have shown that up to 50%-70% of lung cancer patients are complicated by lung infection during the course of the disease (87). Wessels et al. ‘s study on children showed that the risk of TB among children with malignant tumors was indeed higher than that of ordinary children, and the risk ratio reached 11 times (88).

#### 3.2.2 Chemotherapy

Platinum-based chemotherapy is recognized as the standard treatment for patients with stage II and III non-small cell lung cancer (NSCLC) and is often considered for stage IB patients with tumors ≥4cm (87). Chemotherapy drugs have an effective killing effect on tumor cells, but they also affect the immune system of patients. Investigation in TB endemic areas found that TB infection was common in systemic chemotherapy (89). On the one hand, chemotherapy can produce immunosuppressive effects on cellular immunity and immunoglobulin, affecting the normal immune function of patients. Chemotherapy, on the other hand, can cause neutropenia and leukopenia by inhibiting bone marrow function, leading to a state of severe immune deficiency with symptoms such as cough, hypoxia, dyspnea, and fever. In addition, although chemotherapy-induced pulmonary toxicity is rare in clinical practice, it can be manifested as hypersensitivity reaction, interstitial pneumonia, non-cardiogenic pulmonary edema, pleural effusion, obliterated bronchiolitis, tissue pneumonia and progressive pulmonary fibrosis. The above diseases all affect the normal immune defense function of the normal lung, thereby increasing the susceptibility of the lung to bacteria including MTB (90). Clinically, there have been reports of patients with non-small cell lung cancer infected with MTB after receiving standard chemotherapy based on bisplatin and pulmonary TB during postoperative chemotherapy for gastric cancer (91, 92). Another study, which recruited 1,809 cancer cases and 1,809 control subjects and followed them for 3 years, showed that immunosuppression of cancer or anti-cancer chemotherapy increased the risk of TB reactivation, especially in cancer patients with age-cured TB (93).

#### 3.2.3 Immunotherapy

As new immune checkpoint inhibitors continue to enter clinical trials, ICIs is becoming one of the most important immunotherapy therapies. lCIs mainly inhibits immune checkpoint proteins by blocking CTLA-4, PD-1 and PD-L1. At present, immunotherapy of PD-1/PD-L1 has significantly prolonged the survival of patients with advanced lung cancer, and has become the standard treatment choice for the first and second line of advanced lung cancer (94). However, when ICIs are selected, they also affect immune function, which may increase susceptibility to MTB infection. There are two possible reasons for this: First, because ICIs works by blocking CTLA-4, PD-1, and PD-L1 to inhibit immune checkpoint proteins, model studies in knockout mice have demonstrated that conditions in which CTLA-4 and PD-1 are absent may lead to imbalanced immune homeostasis, resulting in reduced host immunity (95–97). Studies have shown that immunocompromised people with latent infections have a higher rate of active TB. Therefore, ICIs leads to a decrease in the body’s immunity, which may be one of the reasons for an increased susceptibility to MTB infection. In addition, pneumonia caused by ICIs is the most common pulmonary toxic reaction, which may also be one of the reasons for the increased susceptibility to MTB infection (98–100). Macrophage apoptosis plays an important role in pneumonia. Through the mutual influence and stimulation of inflammation and apoptosis, the apoptosis of macrophages is accelerated while the inflammation is aggravated (101). After MTB enters the body through the respiratory tract, the body mainly plays a defense role through macrophages and other cells. Therefore, apoptosis of macrophages increases the risk of TB and latent TB reactivation. Through comparative analysis of the function of macrophages at different stages of pneumonia in mice, it was found that macrophages’ ability to phagocytic bacteria was still poor several weeks after the inflammation subsided (102).

## 4 Progress in diagnosis and treatment of TCWT

### 4.1 Progress in the diagnosis of TCWT

At present, the research on the diagnosis technology and treatment plan of TCWT is limited by the variety of tumor types and the uncertain site of TB, and there is no standardized detection means and diagnostic technical guide for patients with TCWT. There are differences in the pathogenesis of TB combined with different tumors, which may lead to differences in detection and diagnosis techniques. For example, early clinical symptoms and CT results of TB and lung cancer are similar, and TB detection technology plus conventional detection technology cannot accurately diagnose (103–105).

Studies on the differential diagnosis of TB combined with malignant tumor FDG-PE/CT carried out in South Korea, Singapore and other countries have shown that FDG-PET/CT has a good diagnostic effect on lung cancer, lymph node and other malignant tumors. Meanwhile, FDG-PET/CT can be used for targeted screening of patients with latent TB infection before immunosuppressive therapy. A useful tool for assessing and excluding active disease sites (104, 106–108). Su Hongjian et al. detected the expression levels of LINC00665 and miR-589-3p in the serum of participants by real-time fluorescent quantitative PCR. Combined diagnosis has better diagnostic value for lung cancer complicated with TB than single detection (109). A study on the use of tumor biomarkers in the diagnosis of pulmonary TB and lung cancer found that the combined detection of CEA, CYFRA21-1 and NSE had diagnostic value for high-risk lung cancer patients with pulmonary TB, with a specificity of 89.9% and a sensitivity of 94.9%. ROC curve analysis showed that CEA+ CYFRA21-1+ NSE had the highest diagnostic accuracy (AUC=0.972) (103). In addition, Han Dongmei et al. used chemiluminescence immunoassay to detect tumor markers in participants. Serum tumor markers combined with CT have a good diagnostic effect on lung cancer complicated with TB (110). Xu Yang et al. used electrochemiluminescence to rank the clinical diagnostic value of patients, from low to high, CEA, CA125, CT and combined diagnosis, among which CT combined with CEA and CA125 in the diagnosis of lung cancer combined with TB had better clinical value (107).

In recent years, RNA and proteomics technologies have also been widely used in the diagnosis of TB and tumor (111–113). Liu et al. used miRCURYTM LAN microRNA to identify fresh peripheral blood mononuclear cells (PBMCs) from microscopic smear-positive pulmonary TB patients and healthy people, and miRNAs in patients with active TB were significantly up-regulated compared with healthy people (114). Other scholars can obtain Ts-RNA from sncRNAs of fresh peripheral blood mononuclear cells (PBMC), which is helpful for early diagnosis of lung cancer and TB (112, 115). At the same time, some studies have applied miRNA as a new biomarker to the diagnosis and prognosis of cancer diseases, and achieved good results (116). This study suggests that RNA biomarkers can be used as a new diagnostic technique in patients with TCWT. Sun et al. used proteomic techniques to compare the proteomic features and plasma protein biomarkers of patients with TB (n=15), patients with latent TB (n=15), and healthy people (n=15). A total of 31 overlapping proteins with significantly different expression levels were identified in patients with PTB compared with LTBI and healthy people. Among them, the diagnostic model composed of α1-antichymotrypsin (ACT), α1-acidic glycoprotein-1 (AGP1), and e-cadhrin (CDH1) had a sensitivity of 75.0% (21/28) and 81.8% (27/33) for PTB and lung cancer, respectively (111). This also suggests that protein can be used as a new diagnostic technology for patients with TCWT, and provides a possibility for optimizing and improving diagnostic technology for subsequent patients with TCWT.

### 4.2 Progress in diagnosis of TCWT

The current anti-TB treatment mainly applies WHO TB guidelines to patients with active TB, and the combination of isoniazid, rifampicin and pyrazinamide is used for 6-9 months (117). If patients are resistant to drugs, the medication regimen needs to be adjusted, and 2-5 sensitive or unused anti-TB drugs should be selected. The whole process of supervised chemotherapy management was implemented to complete treatment for 18-24 months (118, 119). Relatively speaking, cancer treatment means are more diversified, including surgery, chemotherapy and radiation therapy, targeted therapy, immunotherapy, cell therapy and other therapies, which can bring significant benefits to patients (120, 121).

Studies have shown that anti-TB drugs (isoniazid) can cause the decrease of white blood cells and platelets, resulting in liver damage, and cause chemotherapy response in tumor patients, which may act as a suppressant of the therapeutic effect (122, 123). Another study has shown that when chemotherapy and anti-TB therapy for malignant tumors are carried out simultaneously, the treatment of patients is effective and safe (124). It is suggested that the treatment of patients with TCWT is more complicated and uncertain, and the etiology, pathogenesis and therapeutic drug interaction of the two diseases should be comprehensively considered in the formulation of treatment plan (89, 124). For patients with latent TB complicated with lung cancer, anti-TB therapy is generally not required, but only anti-tumor therapy. For patients with active TB, anti-tumor chemotherapy and anti-TB therapy are required (121). Patients with urinary TB complicated with bladder cancer are prone to misdiagnosis or missed diagnosis, which is likely to delay the treatment time and lead to inappropriate treatment options (125). For patients requiring surgical resection of the tumor, preoperative tumor chemotherapy and anti-TB treatment should be synchronized, and anti-TB drugs should be continued after surgery (126), which is conducive to enhancing the therapeutic effect.

In a study of mycobacterium TB infection in mice, mice co-expressed other inhibitory receptors (including PD-1) and were effectively treated with anti-TIM-3 monoclonal antibodies against TB, suggesting that targeting TIM-3 may have significant therapeutic effects in both TB and lung cancer patients (127). In addition, cytotoxic T lymphocyte-associated antigen-4 (CTLA-4), lymphocyte-activating gene-3 (LAG-3), and glucocorticoid-induced TNF receptor (GITR) have gradually become a hot spot for drug therapy to find new targets for TCWT (128). For TCWT, the interaction between drugs should be considered when the anti-TB treatment is synchronized with the targeted therapy of tyrosine kinase inhibitors (TKI). For example, rifampicin can accelerate the metabolism of TKI drugs such as gefitinib, while isoniazid has an inhibitory effect. In order to improve clinical effect, appropriate targeted drugs or immune drugs should be selected according to the drug sensitivity results of patients (129).

In recent years, people have paid more and more attention to the immune evasion of tumor cells and the immunotherapy of Mycobacterium TB, and immunotherapy has gradually become the main treatment methods in the field of tumor, and immune checkpoint inhibitors (ICI) and PD-1/PD-L1 inhibitors have been applied to the treatment of tumor patients with TB (130, 131). Studies have shown that PD-1 is highly expressed in pathological sections of patients with TB complicated with lung cancer (132). Blocking PD-1 may activate T cell function and enhance immune response to tumor and MTB, and PD-1 blocking therapy has important clinical significance in improving the prognosis of patients with TCWT. At the same time, patients with tumor combined with TB should be cautious about using immune checkpoint inhibitor (ICI) or PD-1/PD-L1 treatment. Recent evidence suggests that tumor patients treated with immune checkpoint inhibitors (ICI) may promote TB reactivation or accelerated progression of TB infection (133). Another literature review discussed the incidence of TB caused by PD-1/PD-L1 therapy in tumor patients. Compared with tumor patients receiving PD-1/PD-L1 blocking therapy, the incidence of TB was 35 times that of the general population, reaching every 2,000 cases/100,000 people (134). Clinical use of PD-1/PD-L1 inhibitors may significantly increase the risk of TB reactivation and death in patients (135). Based on this, in clinical practice, clinicians should take comprehensive consideration and risk assessment to develop immunotherapy programs for TCWT, so as to improve the quality and safety of patient diagnosis and treatment. In summary, in the clinical treatment of patients with TCWT, it is necessary to consider the comorbidities to develop joint treatment strategies, and select appropriate treatment methods to play a synergistic or positive role in TB and tumors.

At present, a number of nanomedicine (preparations) have been approved for the treatment of advanced non-small cell lung cancer and other tumors (136, 137). Studies have also shown that nanoparticles have a higher biological distribution in the tumor region and a stronger tumor inhibitory effect (138). In addition, nanotherapy has also been applied to anti-TB therapy, and the nano drug delivery system can further improve the efficacy of anti-TB drugs (139, 140). A variety of nanotechnology has been carried out to study the diagnosis and treatment of TB, and the research results show that the efficacy is good (141–143). Nanotechnology offers more possible strategies for the diagnosis and treatment of TCWT. Currently, nano-dose inhalers have been developed for the treatment of lung cancer and TB, and can also be applied in patients with TB and lung cancer (144). In the future, these new technologies will play an important role in the treatment of patients with TCWT, especially for patients with TB combined with malignant tumor, which will help improve the treatment effect of patients and bring greater benefits to patients.

## 5 Summary

With the progress of science and technology and the diversification of treatment methods, more and more diagnostic methods and advanced treatment technologies have been applied to clinical diagnosis and treatment. The diagnosis, treatment and prevention of TCWT should also be forward-looking, and the application of new diagnostic technologies such as single-cell technology and proteomics in the diagnosis of TB and tumor patients should be actively explored. In the treatment of TB and cancer patients, it is also necessary to actively explore the clinical practice of advanced therapies such as cell therapy and nanotechnology in their diagnosis and treatment, and carry out efficient scientific treatment technologies that are leading, improving and popular. Through systematic literature analysis, we mainly studied the pathogenesis, mechanism, diagnosis and treatment of patients with TCWT. The results showed that TB and tumors interact and influence each other, suggesting that TB and tumor should be diagnosed and treated from a holistic perspective of comorbidities. Integrated and individualized treatment protocols should be developed for patients with an eye to effectively improve the therapeutic effects, and provide ideas for the follow-up clinical research of TCWT, the development of diagnostic technology and the improvement of integrated treatment protocols.

## Author contributions

CW: Data curation, Formal Analysis, Investigation, Writing – original draft. R-QZ: Writing – review & editing. G-ZH: Funding acquisition, Supervision, Writing – review & editing.
